# Supplementary material for: The interactome of intact mitochondria by cross-linking mass spectrometry provides evidence for coexisting respiratory supercomplexes
Source: Mol Cell Proteomics. 2017 Dec 8;17(2):216–32. doi: 10.1074/mcp.RA117.000470 (PMC5795388; doi:10.1074/mcp.RA117.000470)
Supplement: Supplemental Data [file supp_RA117.000470_133922_0_supp_23978_fzffwf.zip › spectra_annotation/mito_spectra_annotation/147-1-13-1-6-1.pdf]

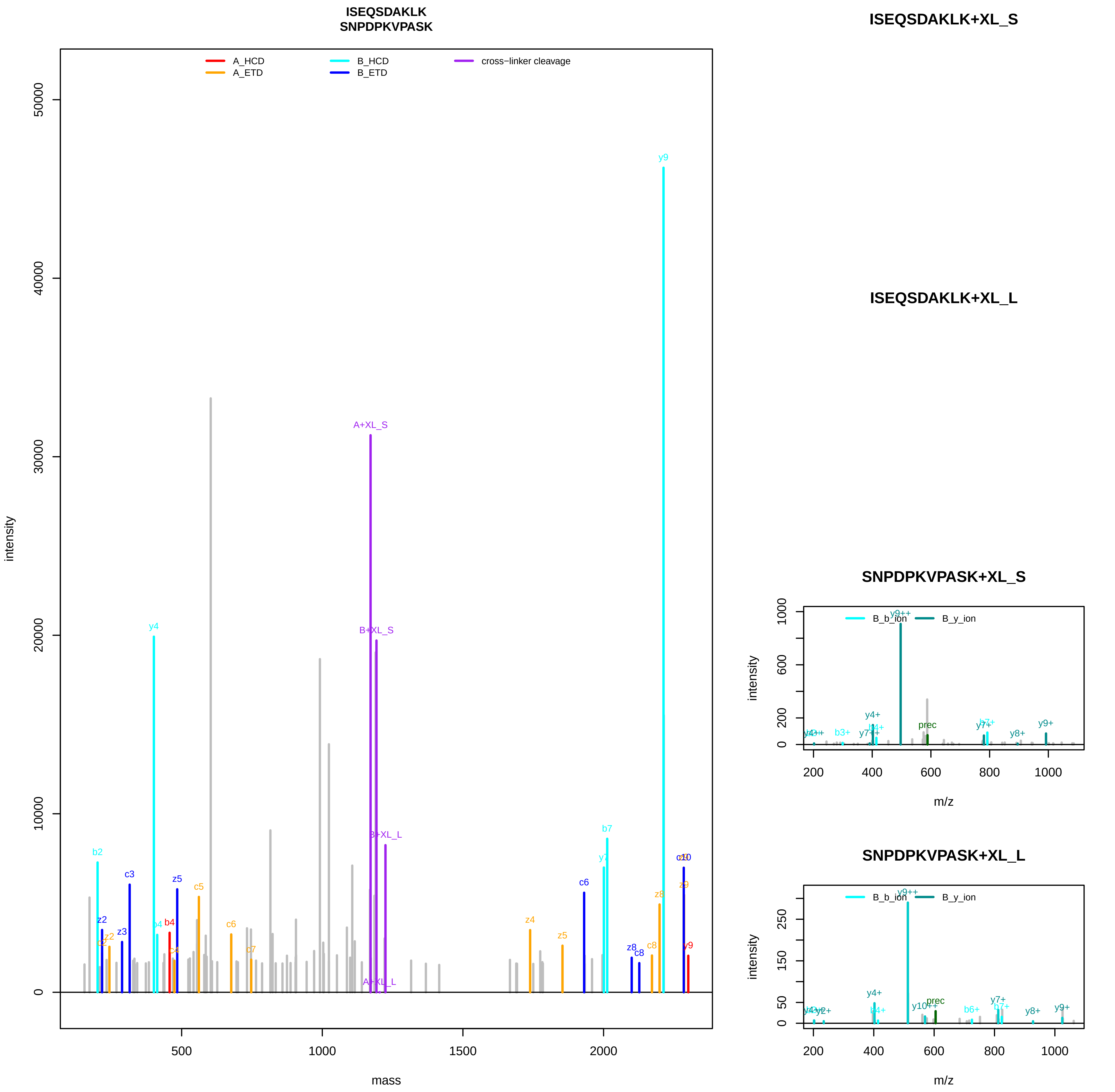
ISEQSDAKLK
SNPDPKVPASK
A_HCD
A_ETD
B_HCD
B_ETD
cross−linker cleavage
intensity
mass
0
10000
20000
30000
40000
50000
500
1000
1500
2000
y9
A+XL_S
B+XL_S
B+XL_L
A+XL_L
y4
b2
c3
z5
c5
b4
z2
z3
c6
c7
z4
z5
c6
y7
b7
z8
c8
c8
z8
c10
z9
y9
ISEQSDAKLK+XL_S
ISEQSDAKLK+XL_L
SNPDPKVPASK+XL_S
B_b_ion
B_y_ion
y9++
y4+
b3+
b4+
y7++
prec
y7+
b7+
y8+
y9+
intensity
m/z
0
200
600
1000
200
400
600
800
1000
SNPDPKVPASK+XL_L
B_b_ion
B_y_ion
y9++
y4+
b4+
y10++
prec
b6+
y7+
b7+
y8+
y9+
intensity
m/z
0
50
150
250
200
400
600
800
1000
